# Effects of White Rice, Brown Rice and Germinated Brown Rice on Antioxidant Status of Type 2 Diabetic Rats

**DOI:** 10.3390/ijms131012952

**Published:** 2012-10-10

**Authors:** Mustapha Umar Imam, Siti Nor Asma Musa, Nur Hanisah Azmi, Maznah Ismail

**Affiliations:** 1 Laboratory of Molecular Biomedicine, Institute of Bioscience, University Putra Malaysia, UPM Serdang 43400 Serdang, Selangor, Malaysia; E-Mails: mustyimam@gmail.com (M.U.I.); nurhanisahazmi@gmail.com (N.H.A.); 2 Department of Nutrition and Dietetics, Faculty of Medicine and Health Sciences, University Putra Malaysia, UPM Serdang 43400 Serdang, Selangor, Malaysia; E-Mail: ana_insyirah85@yahoo.co.uk

**Keywords:** antioxidants, diabetes, electron spin resonance, germinated brown rice, white rice, nutrigenomics

## Abstract

Oxidative stress is implicated in the pathogenesis of diabetic complications, and can be increased by diet like white rice (WR). Though brown rice (BR) and germinated brown rice (GBR) have high antioxidant potentials as a result of their bioactive compounds, reports of their effects on oxidative stress-related conditions such as type 2 diabetes are lacking. We hypothesized therefore that if BR and GBR were to improve antioxidant status, they would be better for rice consuming populations instead of the commonly consumed WR that is known to promote oxidative stress. This will then provide further reasons why less consumption of WR should be encouraged. We studied the effects of GBR on antioxidant status in type 2 diabetic rats, induced using a high-fat diet and streptozotocin injection, and also evaluated the effects of WR, BR and GBR on catalase and superoxide dismutase genes. As dietary components, BR and GBR improved glycemia and kidney hydroxyl radical scavenging activities, and prevented the deterioration of total antioxidant status in type 2 diabetic rats. Similarly, GBR preserved liver enzymes, as well as serum creatinine. There seem to be evidence that upregulation of superoxide dismutase gene may likely be an underlying mechanism for antioxidant effects of BR and GBR. Our results provide insight into the effects of different rice types on antioxidant status in type 2 diabetes. The results also suggest that WR consumption, contrary to BR and GBR, may worsen antioxidant status that may lead to more damage by free radicals. From the data so far, the antioxidant effects of BR and GBR are worth studying further especially on a long term to determine their effects on development of oxidative stress-related problems, which WR consumption predisposes to.

## 1. Introduction

Chronic hyperglycemia in type 2 diabetes promotes oxidative stress and diabetic complications [1], and may even be involved in pathogenesis of the disease [2]. It causes diabetic retinopathy, neuropathy, nephropathy, and cardiovascular disease [1,3]. These microvascular and macrovascular complications cause severe morbidity and significant mortality and could potentially be prevented if oxidative stress is reduced or reversed in type 2 diabetes. Already, 346 million people were reported to be diabetic as of 2011 and this is projected to double by 2030 [4]. Urgent action is therefore needed to reduce the burden of this disease, especially in developing countries where the increase in its prevalence is expected. Alleviating oxidative stress is one way of lowering the risk of complications in diabetics. In this way, the burden of the disease may be reduced.

Diet promotes oxidative stress in diabetes [5], and it is a challenge to prevent diet-induced oxidative stress [6] because food consumption is necessary for survival. White rice (WR) has a high glycemic index [7] and may result in high oxidative stress and other health risks. Recently, it has been shown to increase the risk of type 2 diabetes [8]. However, it is widely consumed as the staple diet by half the world’s population [9]. An alternative to WR would be to consume brown rice (BR) since it lowers insulin and glycemic indices [10], and may confer other health benefits. As BR is difficult to chew, germinating it may improve texture, palatability and the amount of the bioactive molecules [11]. Germinated brown rice (GBR) is reported to have glucose- and cholesterol-lowering properties [11–14]. Consumption of this rice product instead of WR would provide enormous benefits since it will not have the same health risks as WR, but rather will promote health and reduce disease burden [11].

There are reports of bioactive molecules in BR and GBR having antioxidant potential [15,16], and we hypothesized that BR and GBR as dietary components may improve the antioxidant status in type 2 diabetes. Our aim was to show that the commonly consumed WR may worsen antioxidant status, the consequences of which may be promotion of oxidative stress-related complications in type 2 diabetes, and also show that BR and GBR may not worsen antioxidant status as much as WR. Since overall metabolism is perturbed in type 2 diabetes, we also aimed to study the effects of these rice types on expression of antioxidant genes.

## 2. Results and Discussion

### 2.1. Gamma-Aminobutyric Acid (GABA) and Total Phenolic Contents, and Antioxidant Potentials

GABA was undetected in WR while its content in BR was 0.09 ± 0.02 mg/g of BR. Our GBR variety was found to have high GABA content (0.36 ± 0.04 mg/g of GBR). Additionally, GBR had four-fold higher total phenolic content (TPC) compared to BR and its antioxidant assays showed better results than both WR and BR (Table 1).

It is established that BR has bioactive compounds in its bran layer that confer its functionality. GBR has even higher amounts of the bioactive molecules. However, those responsible for the functional properties of BR and GBR are still the subject of debate, though synergy may play a role. The GABA content of our GBR variety significantly improved, and was higher than that which Roohinejad *et al.* [17] reported for the same rice variety and other varieties germinated over the same 24 h period. Similarly, our results showed higher GABA content than that which Charoenthaikij *et al.* [18] reported, suggesting that our method of germination improved GABA tremendously. GABA is an inhibitory neurotransmitter that normally regulates a wide variety of brain functions [19], and it is likely that it is responsible for other functional roles as suggested by Roohinejad *et al.* [17]. Higher GABA content was associated with better hypocholesterolemic effect of GBR in their study [14]. Also, Nakagawa *et al.* reported that GABA protected the liver and kidneys from oxidative damage in type 2 diabetes and hence could reduce the risks of developing diabetic complications from oxidative stress [20]. Higher GABA content in our GBR variety would be expected to confer higher antioxidant properties than its BR or WR counterparts.

Phenolic content has been linked to antioxidant effects of foods [21]. In his review, Burton-Freeman argued that phenolic-rich foods have high antioxidant potentials and could counterbalance negative effects of pro-inflammatory and pro-oxidative foods. Since high phenolic content in foods will improve antioxidant status, higher phenolic content in BR and GBR coupled with higher antioxidant potential than WR, as suggested by our results, means the likely benefits to be derived from BR and GBR would be more than WR. During polishing of BR, the removal of bran layer means that beneficial effects of BR are lost together with the bran, and WR consumption may not confer the same benefits as would be expected of BR considering its high antioxidant potentials. Interestingly, GBR has higher potentials than BR, and may provide the highest antioxidant effects.

### 2.2. Food Consumption and Glucose Analysis

**Figure 1:**
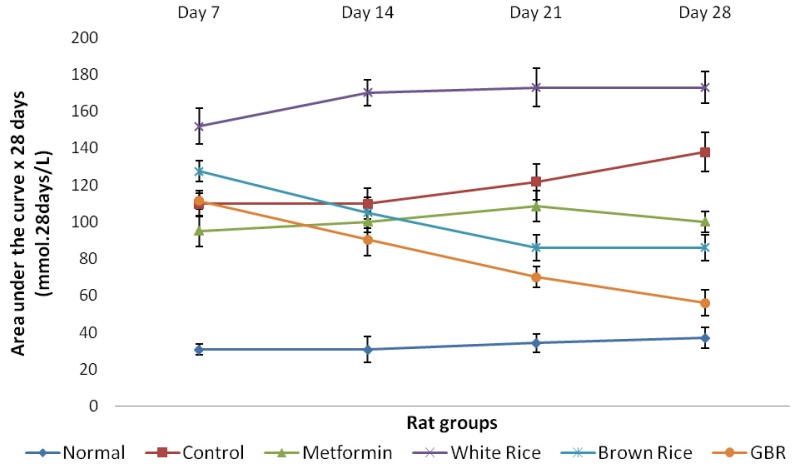
Area under the curve (AUC) for fasting plasma glucose (FPG) over four weeks of intervention; figure shows effect of germinated brown rice (GBR) on AUC of FPG (mmol × d/L) during four weeks of intervention, compared to brown rice (BR), white rice (WR) and metformin (*n* = 5). Groupings are the same as in Table 1.

**Figure 2:**
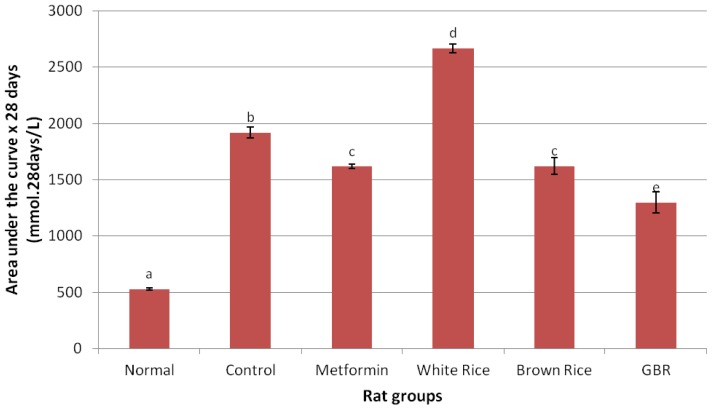
Total area under the curve (AUC) for fasting plasma glucose (FPG) over four weeks of intervention; figure shows effect of germinated brown rice (GBR) on total AUC of FPG (mmol × 28 d/L) over four weeks of intervention, compared to brown rice (BR), white rice (WR) and metformin (*n* = 5). Data used represents mean ± SEM. Groupings are the same as in Table 1.

Table 2 shows baseline parameters including food consumption for each group. Although food consumption was similar for all groups, changes in plasma glucose reflected the effects of the different dietary components given to the different rat groups. Progressive elevation of plasma glucose among diabetic controls possibly reflected the natural history of the type 2 diabetes without any form of treatment. On the other hand, normal rats maintained normal glycemic level throughout the period of intervention and as shown on Figure 1, the area under the curve (AUC) for fasting plasma glucose (FPG) in the normal group was the lowest over a 28-day period of intervention, while WR had the highest and BR and GBR produced lower AUCs than WR. By the end of the experiment, WR group had the highest total AUC (Figure 2), suggesting that high glycemic index of WR maintained an elevated FPG more than all groups including the untreated controls. The total AUC over 28 days for metformin was similar to BR.

Chronic sustained hyperglycemia as a hallmark of type 2 diabetes will continue to worsen without treatment as reflected by AUC of FPG for untreated diabetic (control) group from our study. Worsening hyperglycemia is known to be the primary underlying factor responsible for diabetic symptoms and complications [22]. High glycemic index of WR [7] could have been responsible for the WR group having the highest total AUC throughout the intervention period in the current study. BR has been shown to be a better type of rice due to bioactive compounds, which may lower glycemic and insulin indices compared to WR [12], and as our data suggest, GBR produces an even better effect on glycemic control.

In the current study, we showed that high glycemic index of WR worsened glycemic control in type 2 diabetic rats while BR and GBR produced better glycemic levels. Though a standard hypoglycemic agent [23], metformin produced the same glycemic effect as BR. Our data therefore shows that GBR could produce significant glycemic control, in support of earlier reports [11], better than WR, BR and metformin.

### 2.3. Liver Enzymes, Urea, and Creatinine

At baseline, just after induction of diabetes, creatinine and γ-glutamyltranspeptidase (GGT) were significantly higher in diabetic groups than normal, but other parameters (urea, total antioxidant status (TAS), alanine aminotransferase (ALT) and aspartate transaminase (AST) were not significantly different between groups (Table 2). Elevated creatinine levels among diabetic control, metformin, WR, and BR groups likely suggested ongoing damage to the kidneys. The GBR group, however, did not have as much serum creatinine after 28 days as the other diabetic groups.

At the end of four weeks of intervention, control group had significantly increased serum urea compared to normal rats (*p* < 0.05), while WR had the highest elevation among all groups (Table 2). Metformin group did not have as much serum urea as the untreated group, and BR and GBR groups had similar serum urea elevations to diabetic controls. However, there was no statistically significant difference between WR and GBR groups.

Liver enzymes (AST, ALT, and GGT) in the control group were significantly elevated more than normal group (*p* < 0.05), which were either maintained (AST) or reduced (ALT and GGT). The enzymes were also elevated in WR group. Pattern of liver enzyme changes in the metformin and BR groups were similar, with elevations in both ALT and AST and reductions in GGT. The GBR group had a reduction in liver enzymes.

The liver and kidneys play crucial metabolic roles in the body and are often the targets of insults in type 2 diabetes. Derangements in their function are detrimental to overall functioning of the body. Liver enzymes and serum urea and creatinine are good markers for liver and kidney functions, and suggest the state of these organs. In our study, just after induction of diabetes, biochemical markers (except creatinine and GGT) were not different between diabetic and normal groups suggesting that soon after induction of the disease, the insult to the liver and kidneys may not have been profound enough to produce severe damage. High reserve potentials of liver, and functional versatility of kidneys, may have been responsible for this [24,25], but as diabetes progressed, deterioration in their functions was reflected by our data (Table 2). Higher levels of liver enzymes and serum urea and creatinine were indicative of liver and kidney damage in control and WR groups. Also, metformin and BR were not very effective in preventing the deterioration of kidney and liver function as suggested by the rising biochemical parameters. The reduced liver enzymes in the GBR group, however, may have been indicative of hepatocytes’ protection and/or regeneration. Usuki *et al.* [26] reported that GBR—as a result of its steryl glucoside content—could cause regeneration of sodium potassium adenosine triphosphatase (Na^+^/K^+^ ATPase) and homocysteine thiolactonase (HTase) enzymes in type 2 diabetes, potentially reversing neuropathy and oxidative changes on biomolecules. Our results also indicate that GBR reverses and/or prevents elevations of serum creatinine that is normally seen in diabetic kidney damage, suggesting it could restore function towards normal or protect the kidneys from damage. Not surprisingly, our data show that WR, which has low antioxidant potentials (Table 1) does not prevent the deterioration of kidney and liver functions in type 2 diabetes while GBR produces improvements to varying degrees and BR produces only a modest improvement with better outcome than WR. Higher phenolic and GABA contents, and higher antioxidant potentials of our GBR variety over the WR or BR varieties may have been responsible for the better outcomes recorded for the GBR group for both liver and kidney function parameters. Synergy as a result of several bioactive compounds may also have been involved.

### 2.4. Plasma Total Antioxidant Status

**Figure 3:**
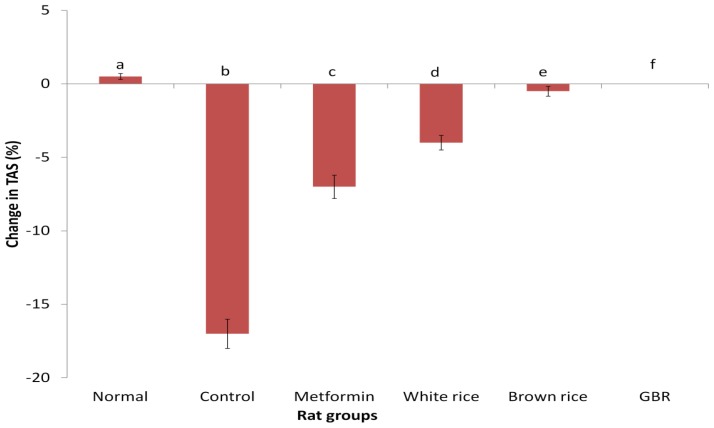
Changes in plasma total antioxidant status (TAS) after four weeks; figure shows effect of germinated brown rice (GBR) on TAS in type 2 diabetic rats after four weeks of intervention, compared to brown rice (BR), white rice (WR) and metformin (*n* = 5). Data used represents mean ± SEM for each group. Bars with different letters are significantly different (*p* < 0.05). Groupings are the same as in Table 1.

Baseline and final measurements for TAS are shown on Table 2, and Figure 3 shows its changes over four weeks of intervention. The TAS for control group was increased while those for control, WR, and metformin groups were reduced. The TAS for the BR and GBR groups did not show much perturbation, and were significantly better than the control, metformin and WR groups (*p* < 0.05). The higher values for BR group over GBR may have been due to higher baseline values, and as the change in TAS suggests, both BR and GBR were able to stabilize their TAS values, with evidence of downward and upward trends for BR and GBR, respectively (Figure 3). As previous data showed, WR caused a significant reduction in TAS compared to BR and GBR, consistently suggesting that WR produces worse metabolic outcomes than both BR and GBR.

Oxidative stress plays significant role in the development and progression of diabetes as well as its complications [1,2], and antioxidants may reduce its level, potentially preventing or decreasing the burden of diabetes. Diet-induced oxidative stress secondary to high glycemic load [6] may have a role in lowering antioxidant status in diabetes as shown from our results for diabetic controls. Also, modest improvements in glycemic control may reduce oxidative stress in diabetes as suggested by TAS in the metformin group. Lowering glycemia may have improved the ability of the rats to produce more antioxidants that removed excess free radicals. For the same reason, WR was expected to show marked reduction in TAS. The fact that WR did not produce as much reduction as expected (Figure 3), suggests the TAS for WR could have been influenced by a number of adaptive mechanisms such as mitochondrial homeostasis [27], leading to a temporary boost in endogenous antioxidant production. Maintenance of TAS by BR and GBR suggests that the 2 rice types were able to replenish the supply of antioxidants that maintained the TAS and/or prevented its deterioration unlike in the case of WR. This effect of BR and GBR in comparison to WR on TAS may have been as a result of the higher antioxidant potentials of BR and GBR. Put together, our findings indicate that better antioxidant status in type 2 diabetic rats may accompany improved glycemic control, and potentially reduce the risks of developing oxidative stress-related diseases.

As can be recalled, WR worsened liver enzymes more than BR and GBR. The liver, as the main “biochemical factory” of the body, is largely responsible for a major aspect of intermediary metabolism and many other functions. The ability of liver to function properly is gradually compromised as type 2 diabetes worsens, and deterioration in TAS for the WR group is indicative that derangement in liver function may have been responsible for the liver’s inability to continue producing endogenous antioxidants. Less damage to the liver due to BR or GBR was likely responsible for their better outcomes.

### 2.5. Hydroxyl Radical Scavenging Capacity of Liver and Kidneys

The liver hydroxyl radical (OH^•^) scavenging capacity of the control group (33.8 mM DMSO equivalent) was lower than that of the normal group (79.8 mM DMSO equivalent) (Table 3). Additionally, the scavenging potentials of the metformin, WR, and GBR groups were similarly lower than the normal group though not significantly different from the control group. Feeding with BR reduced the radical scavenging activity unexpectedly lower than WR. Kidney OH^•^ scavenging capacity was similarly reduced in the diabetic control group compared to the normal (42.8 mM DMSO equivalent compared to 68.8 mM DMSO equivalent). Metformin showed lower scavenging activity (16.8 mM DMSO equivalent) while the WR group lost their kidney scavenging activity entirely. The BR and GBR groups maintained their radical scavenging activities similar to the normal group.

Excess OH^•^ is common in diabetes [1,3] and may damage liver and kidneys, as indicated by the OH^•^ scavenging activity in our study. Accumulated radicals overwhelm functioning capacity of the liver and kidneys with dire consequences. Improved ability of the kidneys to scavenge free radicals, likely due to high amounts of bioactive compounds especially GABA and antioxidant contents, suggests that kidney-specific oxidative stress may be reduced by these bioactive compounds, which were found to be more in our BR and GBR varieties than their WR counterpart.

### 2.6. HEPG2 Antioxidant mRNA Expression

**Figure 4:**
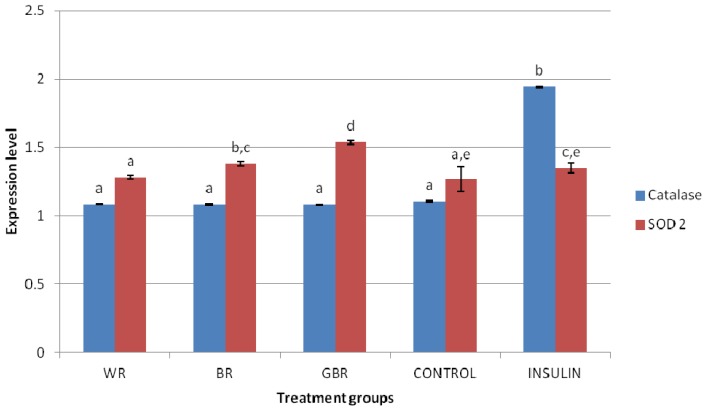
Changes in expression of catalase and superoxide dismutase genes following treatment with ethanolic extracts of germinated brown rice (GBR), brown rice (BR) and white rice (WR); figure shows effect of 70% ethanolic extracts of GBR, BR and WR on expression of catalase and superoxide dismutase (SOD) 2 genes in HEPG2 cells, compared to non-treatment and insulin treatment (*n* = 4). Data used represents mean ± SEM for each group. Bars representing the catalase or SOD2 gene with similar letters are not significantly different (*p* > 0.05).

The expression of antioxidant genes was also studied using an in vitro model to determine if any nutrigenomic mechanisms are involved in BR and GBR’s effects on antioxidant status. The mRNA levels of SOD 2 in the untreated and insulin-treated groups were not significantly different (*p* > 0.05), as shown on Figure 4. The figure also shows that expression of the SOD 2 gene in HEPG2 cells treated with 50 ppm of ethanolic extract of WR did not produce any significant change. However, upregulation of the SOD 2 gene was observed by similar doses of BR and GBR. There was no difference between mRNA levels of HEPG2 cells treated with 50 ppm of WR, BR or GBR extracts, while insulin treatment upregulated the gene significantly. This data shows that nutrigenomic upregulation of the SOD 2 gene may be involved in GBR and BR’s antioxidant effects, and higher amounts of GABA and antioxidant potentials may have contributed to this.

Free radicals generated in biological systems are removed by antioxidants like SOD [29]. It is likely that the antioxidant status due to GBR or BR in type 2 diabetic rats was partly contributed by upregulation of SOD gene. Bioactive compounds like GABA or phenolics may have been responsible. These results support the hypothesis of nutrigenomic interactions between dietary components and genes as a basis for some functional effects of bioactive molecules. Kaput and Rodriguez suggested that this may even be the focus for future management of diet-related chronic diseases [30].

It is common knowledge that GBR grains have high antioxidant potentials just as our results have shown. This potential, hitherto not reported to improve antioxidant status in type 2 diabetes, is shown in this study to likely be responsible for improved glycemic control in type 2 diabetes, and this may translate into reduced risk of developing diabetic complications. Our findings prove that GBR and BR may improve antioxidant status to varying degrees better than WR, which worsens it. The presence of bioactive compounds like GABA and phenolics, and higher antioxidant potentials may have been responsible for these effects. It can be recalled, however, that BR and GBR produced similar outcomes (TAS, kidney hydroxyl radical scavenging activity and expression of catalase gene) in the current study despite higher amounts of bioactive compounds and antioxidant potentials in GBR than BR. It is likely that BR and GBR bioactive compounds have only an “all-or-none” effect on these parameters and others that were similar between the groups. However, it was clear that other efffects of BR and GBR bioactives were exponential (glycemic control and expression of SOD 2 gene), suggesting that higher amounts of bioactive compounds would likely produce even more of the same effect. Also, studies have linked in vivo antioxidant effects of foods to their phenolic content and antioxidant potentials, and this study is in support of that. Other bioactive compounds may also contribute through synergy.

In addition to other risks that have been reported for WR [6,7], our data provides yet another reason why less consumption of WR should be encouraged especially for the rice-consuming populations that use WR as a staple food.

## 3. Experimental Section

### 3.1. Chemicals

All solvents were of analytical grade and were purchased from Merck (Darmstadt, Germany). Alanine transaminase (ALT), γ-glutamyltranspeptidase (GGT), aspartate transaminase (AST), glucose, total antioxidant status, urea, and creatinine kits were purchased from Randox Laboratories Ltd (Crumlin, County Antrim, UK). Ethylenediaminetetraacetic acid (EDTA), Tris-EDTA (TE) buffer solution, 2,2′-azino-bis[3-ethylbenzothiazoline-6-sulphonic acid] (ABTS) reagent, di[phenyl]-[2,4,6-trinitrophenyl]iminoazanium (DPPH) reagent, sodium chloride (NaCl), potassium persulfate (K_2_S_2_O_8_), sodium carbonate (Na_2_CO_3_), γ-aminobutyric acid (GABA) standard, trolox standard, gallic acid standard, Folin–Ciocalteu reagent, and streptozotocin (STZ) were all purchased from Sigma-Aldrich (St. Louis, MO, USA). Hydrogen peroxide (H_2_O_2_) was from Bendosen Laboratory Chemicals (Selangor, Malaysia) and sodium hypochlorite from Dexchem Industries Sdn. Bhd, (Penang, Malaysia). Dimethyl sulfoxide (DMSO), 5-dimethyl-1-pyrroline-*N*-oxide (DMPO) and ferrous sulfate (FeSO_4_) were purchased from Fisher Scientific (Ottawa, Canada), Labotech, Ltd (Tokyo, Japan) and BDH Chemicals (Poole, England) respectively. Nestle fortified milk powder was from Nestle Manufacturing (Selangor, Malaysia) while fine sugar and starch powder were purchased from R & S Marketing Sdn. Bhd. (Selangor, Malaysia). Mazola oil was purchased from Unilever (Malaysia) and standard rat chow from Specialty feeds (Glen Forrest, WA, USA). Metformin was purchased from Pfizer (New York, NY, USA), RCL2 Solution from Alphelys (Toulouse, France), and GenomeLab™ GeXP Start Kit from Beckman Coulter Inc (Miami, FL, USA).

### 3.2. Germination of Brown Rice

PadiBeras Nasional (BERNAS) factory, Sri Tiram Jaya, Selangor provided the white rice (WR) and brown rice (BR) of Malaysian variety (MR220) used in this study. BR was germinated by soaking in 0.1% sodium hypochlorite (1:5, *w*/*v*) and 0.5% hydrogen peroxide (1:5, *w*/*v*) for 30 min and 6 h respectively, and incubated at 37 °C for 18 h. Germination was shown by sprouting, and the final moisture content was 8%–11% after drying at 50 °C.

### 3.3. Gamma-Aminobutyric Acid and Total Phenolic Contents, and Antioxidant Properties

GABA content of the 70% ethanolic extract of GBR was determined as reported by Rozan *et al.* [31]. TPC was determined as detailed by Meda *et al.* [32], while antioxidant assays (DPPH and ABTS) were determined as reported by Kim *et al.* [33]. TPC of BR and WR ethanolic extracts was compared with that of GBR.

### 3.4. Animal Handling, Feeding and Induction of Diabetes

In accordance with the guidelines for the use of animals as approved by the Animal Care and Use Committee (ACUC) of the Faculty of Medicine and Health Sciences, University Putra Malaysia (Project approval number: UPM/FPSK/PADS/BR-UUH/00360), we housed the rats individually (35 male Sprague–Dawley rats; 150–200 g) in plastic cages, in an air-conditioned room (25–30 °C) with a 12/12-h light/dark cycle. After a two-week period of adaptation on normal rat chow and free access to water, five rats were maintained on normal rat chow while the rest were fed with high-fat diet (HFD) for six weeks to induce obesity. The HFD was made up of 40.5% carbohydrate, 16.1% protein, 31.1% fat, 2.5% fiber, and 5.1% mineral and vitamin mix, as formulated in our earlier study [34]. Streptozotocin (STZ) (35 mg/kg b.w.; i.p.) was then injected to induce type 2 diabetes mellitus except in normal rats (5 mmol/L of sodium citrate buffer [pH 4.5]) [35]. Type 2 diabetes was confirmed after two days by fasting plasma glucose of ≥250 mg/dL, and diabetic rats were randomly allocated to six groups of five rats each; control group received HFD, metformin group received HFD and 300 mg/kg/day of metformin [36] while WR, BR, and GBR groups were given HFD in which 50% of the semi-purified diet used to formulate the pellets was substituted with 50% of the respective rice types. Feeding lasted for 28 days.

### 3.5. Plasma Total Antioxidant Status, Glucose, and Liver- and Kidney-Function Tests

Fasting blood samples were collected by cardiac puncture after induction of type 2 diabetes and after 28 days of treatment. Plasma was used for fasting glucose analysis and TAS, while serum was used to measure liver enzymes (ALT, AST and GGT) and kidney function (urea and creatinine levels) using randox analytical kits on an automated chemistry analyzer, Selectra XL (Vita Scientific, Dieren, the Netherlands) according to the kit protocol. Additional blood samples were taken at the end of the 2nd and 3rd weeks for glucose analysis. AUC was calculated as reported previously [37].

### 3.6. Electron Spin Resonance (ESR) Spectroscopy

All rats were sacrificed at the end of the experiment and their organs (liver and kidney) harvested and preserved in RCL2^®^ Solution within 5–10 min of death. Liver and kidney antioxidant status against OH^•^ was measured using an electron spin resonance (ESR) spectrometer (Jeil FA100; Tokyo, Japan). Preserved liver samples (100 mg each) were homogenized in 1 mL normal saline (0.9% Sodium chloride) and centrifuged (Mikro 22R Zentrifugen, Germany) at 10,000 rpm for 10 min. Analyses on ESR were similar to that which was reported by Ismail, Al-Naqeep and Chan [28]. Briefly, 60 μL of the supernatant was added to 40 μL of 0.4 mM DMPO, 37.5 μL of 0.2 mM FeSO_4_, 112.5 μL of 0.2 mM EDTA, and 150 μL of 1 mM H_2_O_2_ at room temperature. The mixture was then inserted (200 μL) into a flat cell (200 pL capacity, quartz form) and measured on the ESR spectrometer. Measurements on the ESR were taken using the following parameters: magnetic field 33.700 ± 5 mT, microwave power 8 MW, modulation frequency 100 KHz, modulation width 0.1 mT, time constant 0.1 s, amplitude 160, and 1 min sweeping time, and DMSO was used as standard.

**Figure 5:**
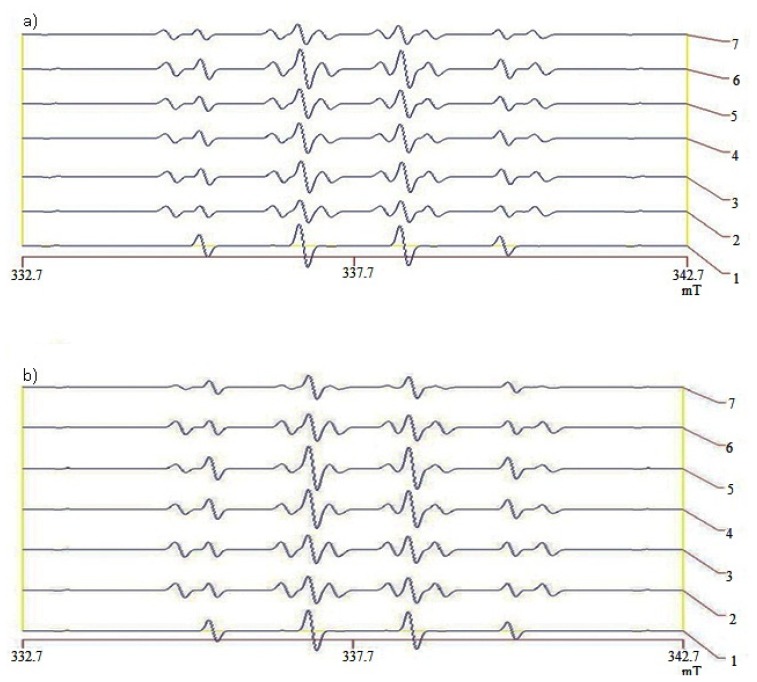
Spectra representing 5-dimethyl-1-pyrroline-*N*-oxide-hydroxyl radical (DMPO-OH^•^) adducts; figure shows electron spin resonance (ESR) spectra of DMPO-OH^•^ adducts representing hydroxyl radical (OH^•^) scavenging activity of (**a**) liver and (**b**) kidneys in type 2 diabetic rats after feeding three different rice types for 28 days. In the presence of DMPO, OH^•^ generated through the Fenton reaction by FeSO_4_ and H_2_O_2_ will form DMPO-OH^•^ adducts that give the signal recorded as spectra on the ESR spectrometer. Scavenging activities (%) were calculated using (*I_o_* − *I*/*I_o_* × 100%) where *I_o_* is the control peak/marker value while, *I* is the peak/marker value for the different rats [28]. Different concentrations (25, 50, 100, 200, and 400 mM) of standard (DMSO) were plotted against scavenging activities (%) to make the standard curve (*y* = 0.4793*x* + 4.5652, *r*^2^ = 0.9959) used to calculate DMSO equivalent scavenging activity. 1 = control, 2 = Normal group, 3 = Diabetic control group, 4 = Metformin group, 5 = White Rice (WR) group, 6 = Brown Rice (BR) group, 7 = Germinated Brown Rice (GBR) group. Other groupings are the same as in Table 1.

FeSO_4_ and H_2_O_2_ from above will generate OH^•^ through the Fenton reaction, which will ultimately form DMPO-OH^•^ adducts represented on ESR spectra (Figure 5), which are reflective of hydroxyl radical scavenging activity. Peak/marker values were used to calculate scavenging activities (%) using (*I_o_* − *I*/*I_o_* × 100%), where *I_o_* is the control peak/marker value while, *I* is the peak/marker value for the different groups [28]. Normal saline was used to obtain control peak/marker value. Different concentrations (25, 50, 100, 200, and 400 mM) of DMSO were plotted against scavenging activities (%) to make the standard curve (*y* = 0.4793*x* + 4.5652, *r*^2^ = 0.9959) used in calculating DMSO equivalent scavenging activities as shown in Table 3.

### 3.7. Cell Culture

We evaluated the effect of the 70% ethanolic extracts (from Section 3.3.) of GBR, BR amd WR on the expression of catalase and SOD 2 genes to determine if any nutrigenomic mechanism was involved in BR or GBR’s antioxidant effects. HEPG2 cells acquired from the American Type Culture Collection (Manassas, VA, USA) were cultured in RPMI 1640 medium supplemented with 10% fetal bovine serum (FBS) and 1% antibiotics (100 U/mL penicillin) in an incubator at 37 °C with 5% CO_2_, following which cell viability was assessed as described by Mosmann [38]. Cells were seeded on a 24-well plate and allowed to attach for 24 h. The cells were serum starved (RPMI 1640 medium with 0.5% FBS and 1% antibiotics) for 12 h and then treated for 24 h with non-toxic doses (50 ppm) of the 70% ethanolic extracts and insulin (100 nM), in a medium that contained 1 μM dexamethasone, 10% FBS and 1% antibiotic.

### 3.8. Hepatic Antioxidant mRNA Expression Analysis

#### 3.8.1. RNA Isolation

RNA from treated HEPG2 was isolated using the GF-TR-100 RNA Isolation Kit (Vivantis, Malaysia) according to the kit protocol.

#### 3.8.2. Primer Design

Primers were designed on the GenomeLab eXpress Profiler software using *Homo sapien* sequence adopted from the National Center for Biotechnology Information GenBank Database [39]. The genes of interest, housekeeping genes and an internal control are shown in Table 4. The forward and reverse primers had universal sequences (tags) in addition to nucleotides that were complementary to the target genes. Primers were supplied by First Base Ltd. (Selangor, Malaysia), and diluted in 1× TE buffer to a final concentration of 500 nM for reverse primer and 200 nM for forward primers.

#### 3.8.3. Reverse Transcription and Polymerase Chain Reaction (PCR)

Reverse transcription and multiplex PCR of RNA samples (50 ng each) were done in an XP Thermal Cycler (BIOER Technology, Hangzhou, China) according to the kit protocol.

#### 3.8.4. GeXP Multiplex Data Analysis

PCR products (1 μL each) from the above reactions were mixed with 38.5 μL of sample loading solution and 0.5 μL of DNA size standard 400 (Beckman Coulter, Inc, Miami, FL, USA) in a 96-well sample loading plate and analyzed on the GeXP machine (Beckman Coulter, Inc, Miami, FL, USA). The results from the machine were analyzed using the Fragment Analysis module of the GeXP system software and then imported onto the analysis module of eXpress Profiler software. Normalization was done with GAPDH.

## 4. Conclusions

In this study we show the evidence that WR worsens antioxidant status in type 2 diabetic rats, while BR and GBR maintain antioxidant status to varying degrees. BR and GBR improved glycemia, TAS, serum creatinine, and radical scavenging potential of the kidneys better than WR. Also, nutrigenomic upregulation of SOD 2 gene may be involved in BR and GBR’s antioxidant effects as a result of higher amounts of GABA and phenolics compared to WR. Although BR and GBR bioactive compounds confer these rice types with their functionality, our findings suggest that some of them may have exponential effects on antioxidant parameters while others may only elicit an “all-or-none” effect. These antioxidant effects of BR and GBR, the exact bioactive compounds responsible for them, and what mechanisms are involved to either produce the exponential or “all-or-none” effects are worth studying further.

## Figures and Tables

**Table 1:** Total phenolic content, DPPH and ABTS antioxidant assays for germinated brown rice (GBR), brown rice (BR) and white rice (WR) extracts.

Extract (70% ethanolic)	TPC (mg GAE/g extract) #	ABTS scavenging activity (mg TEAC/g extract) #	DPPH free radical scavenging assay *,#
WR	0.60 ± 0.45 ^a^	1.78 ± 0.44 ^a^	18.41 ± 0.46 ^a^
BR	3.17 ± 1.68 ^b^	2.67 ± 0.24 ^b^	6.82 ± 0.23 ^b^
GBR	12.01 ± 0.2 ^c^	6.31 ± 0.57 ^c^	5.27 ± 0.17 ^c^

* Expressed as IC50 (mg/mL), calculated from Trolox standard curve (*y* = 0.0087*x* + 4.587, *r*^2^ = 0.9957) in which a lower value suggests a higher scavenging potential.

# Values expressed as mean ± standard deviation. Values with different letters in a column indicate statistical significance at *p* < 0.05. TPC, total phenolic content; ABTS, 2,2′-azino-bis[3-ethylbenzothiazoline-6-sulphonic acid]; DPPH, di[phenyl]-[2,4,6-trinitrophenyl]iminoazanium.

**Table 2:** Baseline parameters for rat groups just after induction of diabetes.

Parameter		Rat group					

		Normal	Control (untreated diabetic)	Metformin	WR	BR	GBR
Glucose (mmol/L)	Baseline	4.6 ± 0.5 ^a^	14.9 ± 2.2 ^b^	14.7 ± 4.1 ^b^	19.1 ± 2 ^b^	18.4 ± 2.8 ^b^	17.3 ± 2.5 ^b^
Food consumption (kcal/100 g body weight/day) *	Baseline	30.5 ± 3.7 ^a^	34.0 ± 6.0 ^a^	30.7 ± 6.0 ^a^	33.2 ± 8.3 ^a^	30.5 ± 6.7 ^a^	33.2 ± 8.3 ^a^
ALT (U/l) *	Baseline	58.32 ± 1.03 ^a^	61.52 ± 2.17 ^a^	61.36 ± 2.47 ^a^	61.52 ± 6.05 ^a^	60.36 ± 4.09 ^a^	58.91 ± 1.34 ^a^
	Final	54.82 ± 1.44 ^a^	62.44 ± 1.75 ^b^	68.11 ± 1.55 ^c^	67.76 ± 2.54 ^c^	71.83 ± 2.58 ^c^	54.20 ± 1.43 ^a^
AST (U/l) *	Baseline	77.22 ± 3.29 ^a^	77.7 ± 2.51 ^a^	75.07 ± 2.35 ^a^	76.64 ± 1.75 ^a^	72.97 ± 3.49 ^a^	74.00 ± 3.38 ^a^
	Final	77.37 ± 1.16 ^a^	84. 69 ± 2.54 ^b^	75.30 ± 2.26 ^a^	81.94 ± 1.23 ^b^	74.21± 2.23 ^a,c^	70.82 ± 2.13 ^c^
GGT (U/l) *	Baseline	2.18 ± 0.43 ^a^	2.70 ± 0.03 ^b^	2.68 ± 0.08 ^b^	3.00 ± 0.38 ^b^	2.83 ± 0.20 ^b^	2.78 ± 0.06 ^b^
	Final	1.92 ± 0.12 ^a^	2.94 ± 0.18 ^b^	2.64 ± 0.16 ^b^	3.24 ± 0.20 ^c^	2.69 ± 0.16 ^b^	2.61 ± 0.16 ^b^
Urea (mmol/L)	Baseline	5.14 ± 0.04 ^a^	5.54 ± 0.45 ^a^	5.32 ± 0.17 ^a^	5.37 ± 0.27 ^a^	5.21 ± 0.43 ^a^	5.23 ± 0.21 ^a^
	Final	5.31 ± 0.06 ^a^	8.42 ± 0.34 ^b^	7.82 ± 0.16 ^c^	9.77 ± 0.39 ^d^	8.18 ± 0.65 ^b,c^	9.20 ± 0.74 ^b,d^
Creatinine (μmol/L)	Baseline	53.86 ± 0.84 ^a^	57. 78 ± 1.26 ^b^	58.55 ± 1.32 ^b^	60.83 ± 5.06 ^b^	58.25 ± 1.67 ^b^	58.32 ± 1.89 ^b^
	Final	53.80 ± 1.20 ^a^	59.92 ± 0.36 ^b^	60.42 ± 0.41 ^b^	62.96 ± 0.78 ^c^	60.76 ± 0.86 ^b^	57.56 ± 0.45 ^d^
TAS (mmol/L)	Baseline	2.12 ± 0.12 ^a^	2.22 ± 0.04 ^a^	2.18 ± 0.04 ^a^	2.14 ± 0.03 ^a^	2.23 ± 0.09 ^a^	2.09 ± 0.09 ^a^
	Final	2.13 ± 0.04 ^a^	1.84 ± 0.02 ^b^	2.03 ± 0.02 ^c^	2.05 ± 0.02 ^c^	2.22 ± 0.01 ^d^	2.10 ± 0.02 ^a^

* Values represent mean ± SEM (*n* = 5). Values with the same letter in any given row are not statistically different (*p* > 0.05). ALT: Alanine transaminase; GGT: γ-glutamyltranspeptidase; AST: Aspartate transaminase; TAS: total antioxidant status. Control and normal groups received high-fat diet (HFD) and normal rat chow respectively while the metformin group received HFD + 300 mg/kg metformin. White rice (WR), brown rice (BR) and germinated brown rice (GBR) groups received HFD in which 50% of the semi-purified diet used to formulate the pellets was substituted with 50% of the respective rice types.

**Table 3:** Liver and kidney hydroxyl radical scavenging activities in type 2 diabetic rats after four weeks of intervention.

Rat Groups	Liver		Kidney	
	
	Scavenging activity (%)	DMSO equivalent	Scavenging activity (%)	DMSO equivalent
Normal	44.5 ± 4 ^a^	79.8 ± 1.2 ^a^	39.0 ± 2 ^a^	68.8 ± 5.2 ^a^
Control	21.5 ± 3 ^b^	33.8 ± 3.2 ^b^	26.0 ± 3 ^b^	42.8 ± 3.2 ^b^
Metformin	22.0 ± 6 ^b^	34.8 ± 2.8 ^b^	13.0 ± 3 ^c^	16.8 ± 3.2 ^c^
White rice	20.5 ± 3 ^b^	31.8 ± 3.2 ^b^	0 ^d^	0 ^d^
Brown rice	13.0 ± 2 ^c^	16.8 ± 5.2 ^c^	39.0 ± 5 ^a^	68.8 ± 0.8 ^a^
GBR	22.0 ± 2 ^b^	34.8 ± 5.2 ^b^	39.0 ± 3 ^a^	68.8 ± 3.2 ^a^

* Values are mean ± SEM (*n* = 5). Values with the same superscript letter in the same column are not significantly different (*p* > 0.05). Hydroxyl radical scavenging activities (%) were calculated using (*I_o_* − *I/I_o_* × 100%) where *I_o_* is the control peak/marker value while, *I* is the peak/marker value for the different rats [28]. Different concentrations (25, 50, 100, 200, and 400 mM) of standard (DMSO) were plotted against scavenging activities (%) to make the standard curve (*y* = 0.4793*x* + 4.5652, *r*^2^ = 0.9959) used in calculating DMSO equivalent scavenging activity. Groupings are the same as in Table 1.

**Table 4:** Gene name, accession number, and primer sequences used in GeXP multiplex analysis of antioxidant genes in HEPG2 cells.

Gene name (Accession number) *	Primer sequences * (with universal tag)	

	Forward	Reverse
Catalase [NM_001752]	AGGTGACACTATAGAATAGCTCAGCTGACACAGTTCGT	GTACGACTCACTATAGGGACCATTCGCATTAACCAGCTT
Superoxide dismutase 2 [NM_000636]	AGGTGACACTATAGAATACAAGCGTGACTTTGGGTCTT	GTACGACTCACTATAGGGAGGGCTTCACTTCTTGCAAAC
Actb [NM_001101] a	AGGTGACACTATAGAATAGATCATTGCTCCTCCTGAGC	GTACGACTCACTATAGGGAAAAGCCATGCCAATCTCATC
GAPDH [NM_002046] a,#	AGGTGACACTATAGAATAAAGGTGAAGGTCGGAGTCAA	GTACGACTCACTATAGGGAGATCTCGCTCCTGGAAGATG
EEF1A1 [NM_001402] a	AGGTGACACTATAGAATACACACGGCTCACATTGCAT	GTACGACTCACTATAGGGACACGAACAGCAAAGCGA
Kanr b		

* based on the *Homo sapien* gene sequences adopted from the National Center for Biotechnology Information GenBank Database [39].

a Housekeeping genes;

b Internal control;

# Normalization gene.
